# Abdominal aortic aneurysms that have ruptured or are at imminent risk of rupture

**DOI:** 10.1590/0100-3984.2017.0096

**Published:** 2019

**Authors:** Ingrid Braga Corrêa, Bruna Leal Torres Alves, Tarcísio Angelo de Oliveira Sobrinho, Laura Filgueiras Mourão Ramos, Renata Lopes Furletti Caldeira Diniz, Marcelo Almeida Ribeiro

**Affiliations:** 1 Hospital Mater Dei - Radiologia, Belo Horizonte, MG, Brazil.

**Keywords:** Aortic aneurysm, abdominal/complications, Aortic aneurysm, abdominal/mortality, Aortic rupture/diagnostic imaging, Abdominal pain/diagnostic imaging, Tomography, X-ray computed/methods, Aneurisma da aorta abdominal/complicações, Aneurisma da aorta abdominal/mortalidade, Ruptura aórtica/diagnóstico por imagem, Dor abdominal/diagnóstico por imagem, Tomografia computadorizada

## Abstract

The rupture of an abdominal aortic aneurysm (AAA) is considered a high-risk surgical emergency, given the catastrophic consequences and high mortality rate. The objective of this pictorial essay is to illustrate the radiological signs that indicate rupture or imminent rupture. To that end, we describe cases treated at our facility and present a brief review of the literature on the topic. The clinical diagnosis of imminent AAA rupture can be difficult, because patients are usually asymptomatic or have nonspecific pain complaints. In the subsequent follow-up, it is possible to identify radiological signs that indicate instability or rupture itself and thus change the prognosis. Computed tomography is the modality of choice for evaluating an AAA and abdominal pain in the emergency setting. It is therefore essential that the radiologist immediately identify the imaging findings that indicate AAA rupture or the imminent risk of such rupture.

## Abstract (pt)

A ruptura de aneurisma da aorta abdominal (AAA) é considerada uma emergência cirúrgica de altíssimo risco, pelas consequências catastróficas e pelo alto índice de mortalidade. Este ensaio iconográfico tem como objetivo ilustrar os sinais radiológicos que indiquem ruptura ou a sua iminência. Utilizamos casos atendidos no nosso serviço e apresentamos breve revisão bibliográfica acerca do tema. O diagnóstico clínico da iminência de ruptura de AAA pode ser difícil, visto que os pacientes são normalmente assintomáticos ou apresentam queixas álgicas inespecíficas. No controle evolutivo, podem ser identificados sinais radiológicos que indiquem instabilidade ou a própria ruptura e, dessa forma, mudar o prognóstico do paciente. Como a tomografia computadorizada é a modalidade de escolha para a avaliação dos AAAs e da dor abdominal no cenário da emergência, torna-se imprescindível o reconhecimento precoce pelo radiologista dos achados de imagem que indiquem aneurismas rotos ou com risco iminente de ruptura.

## INTRODUCTION

In most cases, abdominal aortic aneurysms (AAAs) are asymptomatic and are diagnosed incidentally during imaging examinations performed for other indications. The natural history of an AAA consists in a progressive increase in its diameter and its potential rupture, a medical emergency associated with extremely high mortality and therefore requiring immediate surgical treatment^(^^1^^)^. Early identification of imaging findings indicating the rupture or imminent risk of rupture of an AAA can change the prognosis and ensure more appropriate treatment, making it fundamental that radiologists recognize such changes^(^^1^^,^^2^^)^. Because it is a widely available, rapid imaging method, computed tomography (CT) angiography is the exam of choice in such cases^(^^1^^,^^3^^,^^4^^)^.

The objective of this pictorial essay is to familiarize radiologists with the imaging findings that indicate rupture or imminent rupture of an AAA, using images related to patients examined at our facility. All examinations were performed in multidetector CT scanners: a 160-slice scanner (Aquilion Prime; Toshiba Medical Systems, Otawara, Japan); or a 128-slice scanner (Optima; GE Healthcare, Milwaukee, WI, USA).

A total of 50 adult patients received intravenous injection of low-osmolar iodinated contrast medium, with the aid of an injection pump, at a flow rate of 4.0-4.5 mL/s and an approximate overall dose of 1.5 mg/kg of body weight. Volumetric images were acquired in axial and multiplanar reconstructions, before and after the administration of the contrast, with an emphasis on arterial and venous studies.

## FINDINGS THAT INDICATE AAA RUPTURE

**Figure 1:**
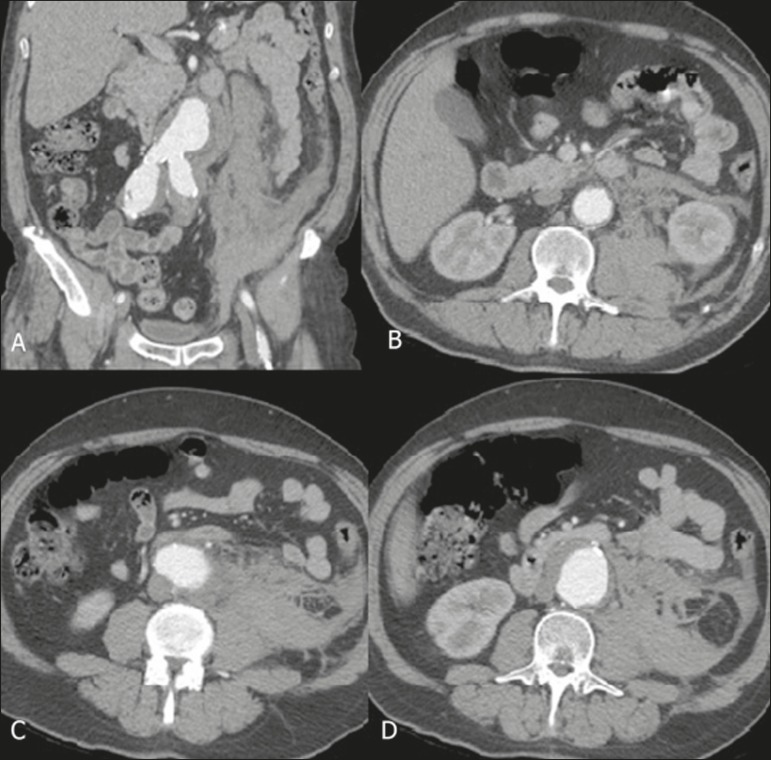
CT angiography with coronal reconstruction (**A**) and axial images (**B,C,D**) in the arterial phase, showing a ruptured AAA, with hematoma and bleeding, extending to the perirenal space and psoas muscle on the left.

The imaging finding most commonly associated with AAA rupture is retroperitoneal hematoma adjacent to the affected aortic segment^(^^5^^)^. That finding translates to a loss of aneurysmal wall integrity and appears on CT as a periaortic focus of soft-tissue density. The hematoma can extend into the pararenal and perirenal spaces (Figure 1), as well as to the psoas muscle (Figure 1) and into the intraperitoneal space. In contrast-enhanced images, active extravasation of the contrast agent can be seen.

**Figure 2:**
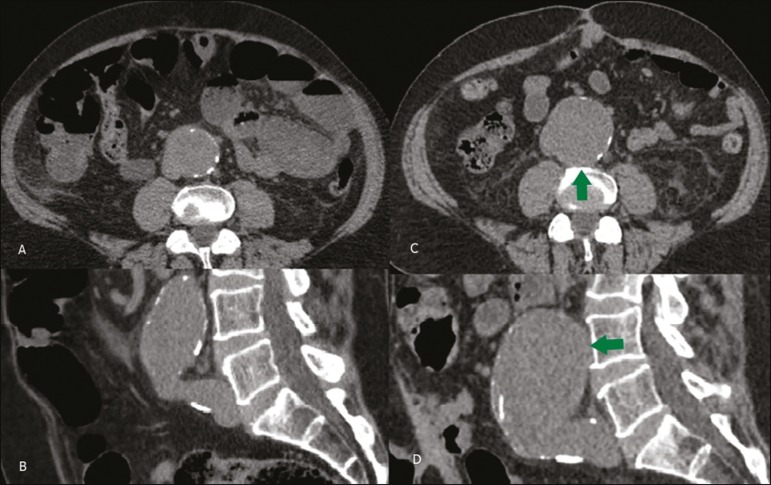
Unenhanced CT scans of a 62-year-old male patient in whom an AAA increased in diameter from 2013 (**A,B**) to 2015 (**C,D**), the increase being accompanied by a loss of the definition of the posterior aortic wall, corresponding to the draped aorta sign (arrow), as shown in axial slices (**A,C**) and sagittal reconstructions (**B,D**).

In the case of a ruptured aneurysm contained, neighboring structures such as the vertebral bodies or adjacent retroperitoneal tissues buffer the hemorrhage and the patient may remain hemodynamically stable^(^^1^^)^. A CT scan of a contained rupture can show the draped aorta sign, in which neither the posterior wall of the aorta nor the periaortic fat plane is distinguishable^(^^1^^,^^5^^)^ (Figure 2).

## FINDINGS THAT INDICATE IMMINENT RUPTURE OF AN AAA

**Figure 3:**
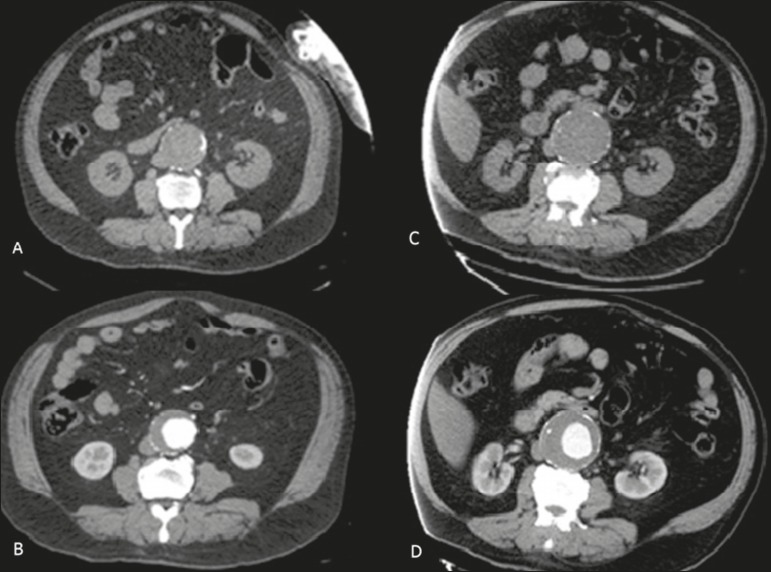
Unenhanced CT scans of a 73-year-old male patient in whom the maximum diameter of an AAA increased by 3 cm over a two-year period, from 5 cm in 2013 (**A,B**) to 8 cm in 2015 (**C,D**), as shown in axial slices (**A,C**) and sagittal reconstructions (**B,D**).

The maximum diameter and growth rate of an aneurysm are the most common predictors of its rupture, underscoring the importance of serial imaging in the follow-up of patients with an AAA^(^^5^^,^^6^^)^. In most cases of typical fusiform aneurysms, a surgical approach is indicated if the aneurysm diameter is > 5.4 cm^(^^6^^)^ or the aneurysm grows by more than 5 mm over a six-month period^(^^6^^,^^7^^)^ (Figure 3).

**Figure 4:**
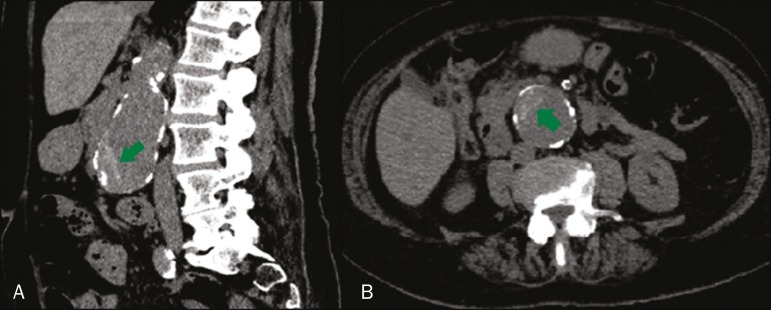
Hyperattenuating crescent sign (arrows), as shown in an unenhanced sagittal CT reconstruction (**A**) and unenhanced axial CT slice (**B**).

The hyperattenuating crescent sign corresponds to a hyperattenuating peripheral area within the wall of the aorta or within a mural thrombus, indicating infiltration of blood from the lumen of the aneurysm into those structures, with consequent weakening of the wall of the aneurysm^(^^1^^,^^5^^)^. The hyperattenuating crescent sign is best visualized on unenhanced CT scans (Figure 4) and is characterized by attenuation greater than that of intraluminal blood.

A focal discontinuity of the parietal circumferential calcification of the abdominal aorta can indicate that an aneurysm is unstable. That is especially relevant when the discontinuity is new or there are new outpouchings^(^^1^^,^^5^^)^.

**Figure 5:**
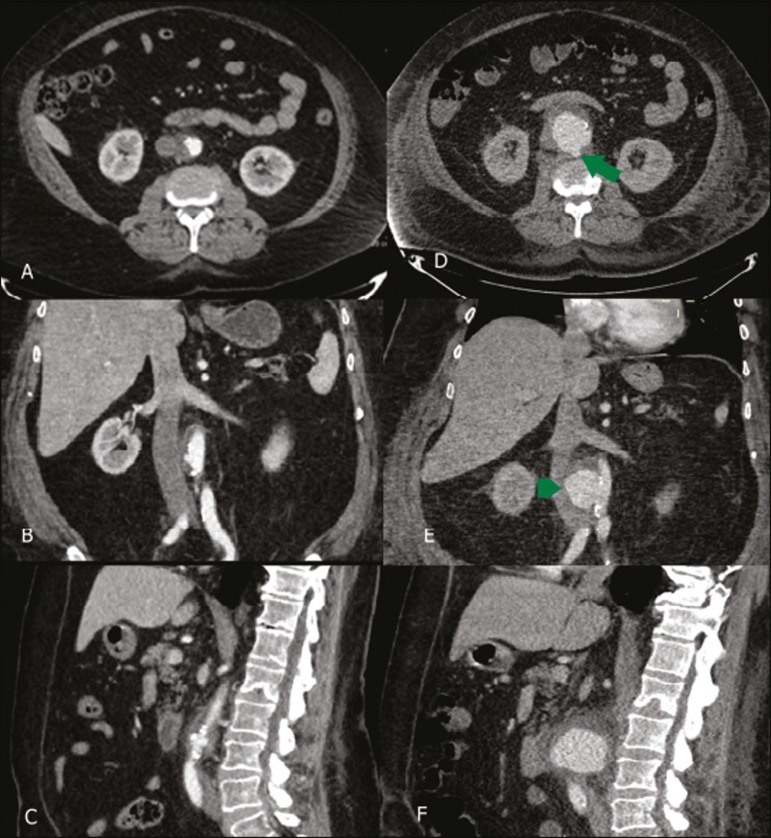
A 69-year-old male patient, complaining of abdominal pain, presenting at 18-day intervals: significant increase in the aneurysm sac and the appearance of a penetrating ulcer (arrow), as shown in axial slices acquired in the arterial phase (**A,D**); new outpouching (arrowhead), as shown in coronal reconstructions acquired in the arterial phase (**B,E**); and periaortic hematoma, as shown in sagittal reconstructions acquired in the arterial phase (**C,F**).

Although less common in AAAs than in thoracic aortic aneurysms, the development of penetrating atherosclerotic ulcers (Figure 5) also indicates that an aneurysm is unstable. The expansion of such ulcers increases the risk of outpouching and rupture^(^^1^^)^.

## CONCLUSIONS

AAA rupture is considered an extreme surgical emergency, because of the catastrophic consequences and the high mortality rate. The clinical diagnosis of imminent AAA rupture can be difficult, given that patients are usually asymptomatic or have nonspecific pain complaints. Because CT is the modality of choice for monitoring the evolution of AAAs and evaluating abdominal pain in the emergency setting, early recognition of CT findings that indicate the rupture or imminent rupture of AAAs is essential and radiologists should therefore improve their knowledge of such findings.

## References

[1] Rakita D, Newatia A, Hines JJ (2007). Spectrum of CT findings in rupture and impending rupture of
abdominal aortic aneurysms. Radiographics.

[2] Wadgaonkar AD, Black 3rd JH, Weihe EK (2015). Abdominal aortic aneurysms revisited: MDCT with multiplanar
reconstructions for identifying indicators of instability in the pre- and
postoperative patient. Radiographics.

[3] Agarwal PP, Chughtai A, Matzinger FRK (2009). Multidetector CT of thoracic aortic aneurysms. Radiographics.

[4] Brown PM, Zelt DT, Sobolev B (2003). The risk of rupture in untreated aneurysms: the impact of size,
gender, and expansion rate. J Vasc Surg.

[5] Gadowski GR, Pilcher DB, Ricci MA (1994). Abdominal aortic aneurysm expansion rate: effect of size and
beta-adrenergic blockade. J Vasc Surg.

[6] Haug ES, Romundstad P, Aadahl P (2004). Emergency non-ruptured abdominal aortic aneurysm. Eur J Vasc Endovasc Surg.

[7] Savarese LG, Trad HS, Joviliano EE (2017). Fistula between the abdominal aorta and a retroaortic left renal
vein: a rare complication of abdominal aortic aneurysm. Radiol Bras.

